# Experience and the nutritional relevance of protein-enriched ice cream in patients with heart failure - A pilot study

**DOI:** 10.1016/j.heliyon.2024.e33661

**Published:** 2024-06-25

**Authors:** Rebecca Gagnemo Persson, Eva Drevenhorn, Helena Rosén

**Affiliations:** Dept of Health Sciences, Faculty of Medicine, Lund University, Lund, Sweden

**Keywords:** Heart failure, Pilot study, Patients' experience, Protein-enriched ice cream

## Abstract

**Background:**

Patients with heart failure have a greater risk of malnutrition and need an enhanced intake of nutrients.

**Purpose:**

The purpose of the study was to describe how patients admitted to a cardiac intensive care unit with heart failure, experience the intake of a protein-enriched ice cream, served as an in-between meal, and to explore this diet supplement's nutritional relevance in these patients.

**Method:**

In the pilot study, interviews, and collection of diaries with both a qualitative and quantitative approach were used. Inductive, qualitative content analysis was performed of the interviews while data from the diaries were analysed with descriptive analysis.

**Results:**

The enriched ice cream supplement was perceived as appealing and tasty despite the patients illness and malaise. Different opinions about consistency were experienced according to the patients’ individual condition and they made further flavour suggestions. The patients ingested between 500 and 2550 ml each of the ice cream during the study period.

**Conclusion:**

The patients’ experiences can be valuable in order to improve the in-between meal/food snack thus improving the nutritional status of weak patients lacking appetite. The method used requires to be further developed to assess nutritional relevance for patients when consuming a protein-enriched ice cream.

## 1 Introduction

One necessity for a good quality of life is a good nutritional status, which has a beneficial effect on both the prevention of disease and medical treatment [1]. Patients who have an increased risk of developing a low nutritional status or malnutrition are those with heart failure, a diagnosis affecting approximately 64 million people worldwide [2]. In Sweden, approximately half a million people are living with heart failure [3] and even though the annual incidence rate has declined over time the prevalence of heart failure has increased, implicating enhanced survival rate. Heart failure is, in Sweden, one of the most common reasons for hospitalization [4] and the most common cause of heart failure is hypertension [5]. In the treatment of the disease, it is, of course, important to address the underlying cause to improve the patient's prognosis and at the same time alleviate symptoms [6].

Heart failure is defined by the European Society of Cardiology (ESC) as “a clinical syndrome characterized by typical symptoms (e.g. breathlessness, ankle swelling and fatigue) that may be accompanied by various signs (e.g. elevated jugular venous pressure, pulmonary crackles and peripheral oedema)”. Individuals with heart failure have an increased risk of developing malnutrition due to several reasons, where one is dyspnoea leading to an increased energy consumption. At the same time, the breathing difficulties make it hard to have a sufficient intake of food. This combination may increase the risk of malnutrition [7]. In heart failure, cytokines are released which trigger a chronic inflammation which in turn may increase the energy consumption, thus enhancing the risk of malnutrition [8].

It has been reported that a loss of appetite is common among patients with heart failure [9] which could be linked to early satiety. In experimental animal studies, levels of the satiety inducing hormone cholecystokinin was elevated in animals subjected to ligation of left anterior descending artery thus inducing a myocardial infarct while the levels of the food-intake stimulating hormone, ghrelin, was lowered. In contrast to the control group, the animals with heart failure lost weight postoperatively, indicating the development of cardiac cachexia [10]. This phenomenon is also seen in humans [11,12]. Despite feeling satiety and a loss of appetite, patients have a longing for food, which leads to feelings of disappointment when the meal does not meet their expectations. Instead of feeling the pleasure and satisfaction of having an enjoyable meal, patients with heart failure may experience that the meal becomes tedious in order to get enough nutrition and energy to cope with everyday life [13]. In the treatment of patients with heart failure, dietary restrictions such as reduced or limited salt intake and fluid restriction of 1.5–2 L per day are common. These restrictions may contribute to the individual not knowing which food that can be prepared and eaten and the experience that the food is not as tasty as it used to be may entail that he or she eats less with increased risk of malnutrition [8].

Malnutrition is proposed to be defined as Body mass index (BMI) < 18.5 kg/m^2^, but also the combined occurrence of more than 10 % unintentional weight loss from usual weight regardless of time or a loss that is more than 5 % over a period of three months associated with at least one of the following: reduced BMI (BMI <20 kg/m^2^ (patient younger than 70 years) or BMI <22 kg/m^2^ (patient older than 70 years), or low fat-free mass index (FFMI, <17 and < 15 kg/m^2^ in men and women respectively) [14]. To cover the daily nutritional and energy needs, around three to five in-between meals are recommended [15]. Malnutrition is estimated to occur in 30–50 % of all individuals in inpatient care and has an extensive impact on health care costs [16]. Malnutrition contributes to longer hospital stays and an increased incidence of complications. Patients who were malnourished during the care period were found to require continued care and attention at home after discharge to a greater extent than patients who were not malnourished [16]. Malnutrition also increases mortality in patients with chronic diseases [17].

In the work of preventing and treating malnutrition, it is pivotal that care is individualized and that the individual's special needs are respected [1]. Since patients with symptoms like tiredness/fatigue, nausea and lack of appetite/anorexia, e.g. patients suffering from heart failure, benefit from small energy-dense meals [18] knowledge may be needed about how this patient group experiences e.g. a protein-enriched ice cream as a healthy in-between meal/food snack.

In health care, malnutrition is assessed based on whether the patient has lost weight involuntarily or if the patient has eating difficulties and if the patient is underweight according to the Body Mass Index (BMI). If an individual is unable to satisfy his or her basic need to get enough fluids and food, it is essential that someone else take on this responsibility, such as a nurse who is, among other things, responsible for the patient's basic need to eat and drink [19].

Person-Centred care is defined as the patient being the focus of care and that the patient is seen as a unique individual with specific needs and values [20]. Patients should have the right to participate in decisions and treatments, as well as to express their own wishes [21]. In caring encounters with patients who suffer from heart failure, they may express preferences of taste and texture on food in general. By asking for and listening to the individual's experiences and wishes, essential information can be generated on various preferences, which can help to improve the in-between meal to be healthier. Previous studies have highlighted the importance of taking into account the individuals' preferences when it comes to texture, flavour and temperature of meals and in-between meals [15,22]. Since patients with heart failure often are seriously ill, with an enhanced need of energy [23] these individuals may have an increased need for nursing on nutrition. In such situations, frequent small energy powered meals, and snacks such as protein-enriched ice cream may be beneficial and may prevent the development of malnutrition. Therefore, we conducted this study about how a protein-enriched ice cream may contribute to an improved nutritional status and diet for patients with heart failure including the patients' preferences regarding different flavours of the ice cream in order to assess the feasibility of this in-between meal as a first step in a clinical nutritional study.

The purpose of the study was to describe how patients admitted to a cardiac intensive care unit with heart failure, experience the intake of a protein-enriched ice cream, served as an in-between meal, and to explore this diet supplement's nutritional relevance in these patients.

Specific research questions

1. What amount of the protein-enriched ice cream did the patients eat?
2. How do patients with heart failure experience the intake of the protein-enriched ice cream?

## 2 Materials and methods

The study was a pilot study with patients admitted to the cardiac intensive care unit at a university hospital in the southern part of Sweden. Inclusion criteria were having heart failure, being over the age of 18 and able to understand, read and write Swedish. Exclusion criteria were acceptance for heart transplantation or intolerance of the ingredients of the protein-enriched ice cream. Patients meeting the inclusion criteria were asked to participate in the study on the day of admittance to the care unit. Eight participants were included after informed written consent between April 2018 and June 2019.

### 2.1 Intervention

The intervention consisted of serving protein-enriched ice cream to patients with heart failure. The first four participants received two servings of ice cream of 200 ml/day, to find out if there was an interest in eating the ice cream at all. The following patients were given free access to the ice cream. Patients received servings of 200 ml of the protein-enriched ice cream as a snack or in-between meal. One serving of 200 ml (100 g) contained 210 kcal, 10.5 g protein, 2.5 μg vitamin D, 0.75 μg vitamin B12 and 9.8 g fat and was available in three different flavours; caramel, vanilla, and lemon. For four to five days or for as long as the patient stayed at the cardiac intensive care unit, it was documented in a diary how many portions of ice cream and what taste they had eaten (Appendix 1). Before discharge from the hospital, the participants were interviewed using a semi-structured interview guide (Appendix 2). The guide contained areas to be covered e.g. how the ice cream was perceived to swallow, any trouble of nausea, burning mouth and throat pain, perceived satiety and preferred flavour of the ice cream, and the patients’ thoughts on having the protein enriched ice cream as a means to improve their diet.

The interviews were audio-recorded, performed by the authors (#1, #2, #3) and lasted between 30 and 60 min. All interviews started with the open question “How did you experience the ice cream?”, followed by questions according to a semi-structured interview guide (Appendix 2). The audio-recorded interviews were transcribed verbatim by the first and last authors (#1, #3) and by two nursing students who were commissioned as a part of their BSc thesis. The two students also performed the content analysis [24], supervised by the third author (#3). The authors read the transcribed interviews several times to get an understanding of the content of the interviews. From the transcribed text, meaning units were picked out and given a code. The codes were then sorted into categories and subcategories.

To answer research question 1 about the amount of ice cream the patients consumed and what that amount gave them nutritionally, the figures from the filled in diaries were summarized, and the mean of the intake of each nutritional factor was calculated.

## 3 Results

The results from the diaries and interviews showed that the experience of eating the protein-enriched ice cream could be compiled into five subcategories and two main categories (Table 1). Quotations are used to illustrate the patients’ experiences and it is presented from which interview each quote derives. The/ … /mark means that words or sentences that are not relevant to the quotation have been omitted.

**Table 1:** An overview of the sub-categories within each main category.

Subcategory	Main category
Taste	Experience of the ice cream
Texture	
Desire of flavour	
Temperature	When feeling ill
Energy and satiety	

### 3.1 Results from the diaries

Patients ate between 500 and 2550 ml over a period of three to eight days (Table 2). This gives an average of 1316 ml in five days or 263 ml per day. During the stay in the intensive care unit where the patients had the ice cream, they ingested between two and 12 portions; the average value then being 7.1 portions.

**Table 2:** Amount of ice cream consumed and amount of nutrients the ice cream contributed to, for four patients who had two portions of the protein-enriched ice cream per day.

Patient	Total amount ice cream, number of days and portions			Total amount of each nutrient/energy during the study via the ice cream				
	Total amount of ice cream (ml)	Total number of days consuming ice cream	Total number of portions	Vitamin D (μg)	Vitamin B12 (μg)	Energy (kcal)	Protein (g)	Fat (g)
Patient 1	2000	5	10	36	11	3000	150	140
Patient 2	2300	5	12	41	12	3450	172.5	161
Patient 3	830	4	6	15	4.5	1250	62.5	58
Patient 4	850	3	5	15	4.5	1275	64	59.5

With the ice cream, the patients had an additional daily intake of vitamin D between 8.93 μg and 45.54 μg, with an average value of 23.5 μg per patient. The daily intake of vitamin B12 was then between 2.68 μg and 13.66 μg with an average of 7.1 μg.

The total amount of calories the patients received with the ice cream was between 750 kcal and 3825 kcal and the protein amount varied from 37.7 g to 191.25 g with an average of 98.7 g. In total the intake of fat varied between 35 g and 178.5 g, with the mean value of 92.1 g (Table 2, Table 3).

**Table 3:** Amount of ice cream consumed and amount of nutrient the ice cream contributed to for four patients who had had several portions of protein-enriched ice cream per day.

Patient	Total amount ice cream, number of days and portions			Total amount of each nutrient/energy during the study via the ice cream				
	Total amount of ice cream (ml)	Total number of days consuming ice cream	Total number of portions	Vitamin D (μg)	Vitamin B12 (μg)	Energy (kcal)	Protein (g)	Fat (g)
Patient 5	2550	8	10	45.5	14	3825	191	178.5
Patient 6	500	5	6	9	2.5	750	37.5	35
Patient 7	1000	5	5	19	5.5	1500	75	70
Patient 8	500	5	3	9	2.5	750	37.5	35

### 3.2 Results from the interviews

#### 3.2.1 Experience of the ice cream

Experiences of ice cream concerning taste, texture and desire of flavour appeared during the interviews.

#### 3.2.2 Taste

The patients expressed different preferences on which flavour, lemon, caramel, or vanilla. The diaries showed that most patients chose the vanilla taste, and in the interviews, it was described as the best one. However, one patient described that the vanilla flavoured ice cream tasted like powder. Regarding the caramel flavour, some patients felt that it was too sweet while others preferred it over the other flavours. Several patients described the lemon flavour as fresh and sour with a lot of taste, while one patient meant that it did not taste like lemon at all but more something in between peach and mango. One patient felt that the other ingredients in the lemon ice cream came through too much.

“*The egg taste came through stongly in the lemon flavour,/ … / but because the lemon taste was weak it felt a bit like beaten egg*” (Interview 7)

Another patient described the lemon flavour as " … positively surprising" (Interview 2) because he usually avoided lemon as he had a lot of stomach acid. One patient also described that it was good to mix the various flavours together. But despite different preferences regarding the flavours, all patients felt that the ice cream was appreciated and tasty in its entirety and some also meant that the ice cream tasted like regular ice cream.

#### 3.2.3 Texture

It was evident that the patients had different preferences about which consistency of the ice cream was best. Most patients were also offered to taste the ice cream as a milkshake (thawed ice cream) during the interview and some experienced it as good and preferred it melted rather than frozen. Others experienced that the melted ice cream made them nauseous, that the thawed ice cream felt “insipid" (Interview 4) and that the ice cream became sweeter when melted. Several experienced the ice cream as cooling and toothsome when it came directly from the freezer, but one of these patients also described that the taste of the vanilla ice cream did not emerge directly if it was too frozen.

” *… and if you wait for example 5 or 8 minutes then it gets to taste best”* (Interview 3)

#### 3.2.4 Desire of flavour

In the interviews, it emerged that the patients wished that there were additional flavours of the ice cream. Some of the desired flavours that appeared several times were strawberry and pear flavours. One patient also wanted a liquorice flavour, and another wanted a punch parfait flavour. Several patients wished that the ice cream was available in flavours such as mango, passion fruit or raspberry which they perceived as a more healthy and fresh flavour.

“ … *but raspberry and blackberry are such that camouflage the taste more and you can reinforce that with let’s say passion fruit because I don’t think you can have pure passion fruit ice cream”* (Interview 7)

One of the patients conveyed his desire for a flavour by sharing a memory from an ice cream he ate when he visited Denmark,

" … it was a vanilla ice cream covered in green juice" (Interview 2).

### 3.3 When feeling ill

When feeling ill, the ice cream was sometimes perceived as a way to get an adequate amount of calories and energy where the temperature of the ice cream could be important especially when feeling nausea, and also if the patients did not experience any hunger; to get energy and satiety, the ice cream was an easy meal.

#### 3.3.1 Temperature

Most patients described the ice cream as cooling and best when it was cold, but some thought that the ice cream was negatively affected by frost in the freezer. The ice cream was easy to swallow. A patient had been in a ventilator and felt a blister in his oral cavity. Despite this and other swallowing problems, he felt that the ice cream was easy to eat.

Some patients stated that the environment and situation during meals are of great importance for appetite. One patient described it as good to be able to eat ice cream when you cannot get out of the room. An advice that emerged was that the ice cream should not be given at a stage where you feel too ill as the ice cream could then be connected to nausea e.g.

*” because when you’re feeling so poorly you don’t feel any hunger either”* (Interview 4)

#### 3.3.2 Energy and satiety

It was stated that food was only eaten to meet the daily calorie intake. The patient did not feel hunger but ate, nonetheless. Ice cream was then an easy way to get enough calories. The ice cream was experienced as much tastier than energy or protein drinks and did not need to be forced down compared to having a protein drink, it was stated. However, one patient thought that a more prominent flavour would have obscured the taste of egg and made the ice cream even more appetizing.

All patients interviewed, expressed that the ice cream gave a high saturation, describing it as powerful, strong, and more nutritious than regular ice cream. Likewise, everyone thought that ice cream was a good in-between meal to improve the diet. Some patients described it as a helpful complement when none of the meals suited their preferences or if the patient was unable to eat solid and/or cooked food due to nausea.

*“ … yeah yesterday evening they served some sort of salmon pudding or what the heck it was and that was not to my liking, so I had a big plate of ice cream instead”* (Interview 5)

## 4 Discussions

In the present study, the researchers first let four participants receive two servings of ice cream of 200 ml/day to find out whether there was any interest in eating the ice cream at all. Following this, the intent was to include additional 15 participants following the protocol for the pilot study. As it was difficult to recruit participants these first four patients as well as the following four were included in this study. This means that the two patient groups in our pilot study were given different conditions and therefore had access to different amounts of ice cream per day, which have affected the result and is a weakness in the study. The result is therefore reported in two different tables according to which group of participants the patients belonged; those who received two servings per day and those who had free access to the ice cream.

During the interviews, it emerged that the participants had eaten the ice cream on more occasions than reported in the diaries. This can be explained by the fact that in many cases the patients were too ill to be able to keep a diary themselves and the responsibility to report the occasions in the diaries was therefore placed on the healthcare staff, who did not follow the instructions correctly. This resulted in the diaries not being complete which is a weakness of the study. The results of the diaries showed that the patients who received free access to the ice cream ate less than those who received two servings per day. This may be because the patients were not asked by the staff to the same extent and did not themselves ask for another portion of ice cream. In the analysis of the descriptive statistics, the authors have been aware that the different patient groups consumed different amounts of ice cream and the results between the two groups can therefore not be compared. In a future study the data collection must be even more closely monitored by the researchers.

The result showed that the patients ingested different amounts of ice cream and thus received different amounts of nutrition. For a more overall picture of the participants' nutritional status and the benefits of the in between meal ice cream we would have needed more outcome measures e.g. the weight of the patients and their daily intake of other foods and drinks. Future more developed studies within this field could provide results that show how the ice cream possibly could contribute to the patients’ nutritional status.

During the analysis, the BSc students worked with an open approach and had a continuous dialogue with one of the researchers (#3). In addition, the students in parts of the content analysis used triangulation with one of the researchers (#3) to prevent the interpretations of the content from being one-sided, which strengthens the credibility [25].

The results showed that patients experienced the protein-enriched ice cream differently due to personal preferences of taste and texture and based on health status. As individuals have different perceptions of their needs and how they can best be met, a responsibility is placed on the nurse to help the patients to express their desires and feelings, which is described as one of the basic needs for achieving health and recovery [19].

Patients with heart failure may experience that food does not taste good [8] but also that their appetite may be affected negatively [26]. The results of the present study show that the patients experienced the protein-enriched ice cream as a good alternative when the food at the hospital did not suit their preferences and several described the ice cream as a better alternative to nutritional drinks. The ice cream was also shown to be a good complement as a dessert. Ice cream can thus contribute to improved food intake and moreover that intake of food is perceived as more satisfactory [13]. As a nurse, it is important to see to the patient's individual needs and to help and support patients who have an insufficient nutritional intake [19].

One way to support the patient's nutritional intake is to offer a protein-enriched ice cream as a healthy food snack or complement to another meal. If the patient has supportive interventions such as parenteral nutrition or tube feeding, it is essential that the nurse has the right competence to support the care of the patient [19]. This kind of ice cream can possibly be a good complement to these measures and an effective way for improved nutritional status.

The result shows that the ice cream meal was perceived to give a high degree of satiety and was considered as a good, tasty, and simple healthy food snack that helped to improve the diet. Since research has shown that patients with heart failure have an increased risk of developing malnutrition due to the increased energy needs [8] ice cream can contribute to meeting the daily energy needs. If the individual has difficulties in meeting the daily energy needs, it is advantageous to eat energy-dense healthy in-between meals in order to get large parts of their daily needs from these meals [14]. In addition to patients' suffering, malnutrition also accounts for a large part of healthcare costs and in this way an improved nutritional status can benefit not only the individual's health but also healthcare and, in the long run, society's costs [16].

In the result, the patients expressed that their really preferred flavours of ice cream were not available which in turn could have further improved their diet. Among other desired flavours were pear, strawberry, liquorice, and passion fruit. By listening to the wishes of the patients, the healthy food snack can be improved, which in turn could contribute to more individuals liking the healthy food snack and choosing to use the enriched ice cream for an improved diet. By listening to the patient's wishes, the patient's participation in decisions and treatments is strengthened, as well as his and her right to express their will and wishes [27].

The individual's state of health emerged to be a factor in how the protein-enriched ice cream was experienced. It turned out that one patient who had problems with his stomach normally avoided the lemon flavour but after having tried it, was positively surprised. Another patient described the ice cream as most beneficial when you are ill, which may be due to that ice cream is easy to eat as it is cold and cooling that triggers the swallowing reflex. As the state of health has a great impact on how food intake is experienced, it is important that the nurse is sensitive to the individual's altered situation and needs in order to be able to help the patient in the best possible way [26].

The sensory experiences such as texture, colour and temperature affect how the individual experiences food intake and everyone has different preferences of texture on food and drink [28]. The protein-enriched ice cream can be eaten immediately when it is taken out of the freezer, but also as a milkshake if it is left for a while and allowed to thaw. The results showed that this enabled the ice cream's consistency to be adapted according to the patient's wishes for an in-between meal. This is beneficial because the presentation of food is important for how good the meal is considered to be [29]. A meal that is carelessly laid out is not considered to taste as good as the same food that is instead presented nicely on the plate [29]. As the protein-enriched ice cream can be adapted according to the patient's wishes regarding presentation and consistency, it contributes to the healthy food snack being served in a way that makes it attractive to the patient.

## 5 Conclusions

An improved nutritional status in patients with heart failure who are malnourished has major benefits on the individual's health and healthcare costs. The results show that the protein-enriched ice cream was perceived as a good, tasty, and satiating healthy food snack that can be used to improve the diet. However, individuals have different preferences for flavour and consistency and by listening to the patient's experiences, the ice cream can be improved so that more people can partake of the healthy food snack. Further studies are needed to investigate to what extent the protein-enriched ice cream, used as an in-between meal, can contribute to malnourished patients' nutritional status. The pilot study has been helpful in realising how such a study can be designed.

## Ethical statement

The study adheres to the declaration of Helsinki (WMA 2000) and was reviewed and approved by Regionala Etikprövningsnämnden Lund (Regional ethics review board in Lund) with the approval number: 2018/4, dated 2018-04-12.

All patients provided written informed consent to participate in the study and for their data to be published. In addition, all patients were also asked for oral consent prior to the interviews.

## Data availability

We find it valuable to share research data as it helps other researchers to evaluate their findings. However, with respect to our informants, who all were seriously ill, the data has been used in confidentiality. The data has been deposited into a publicly available repository.

## CRediT authorship contribution statement

**Rebecca Gagnemo Persson:** Writing – review & editing, Writing – original draft, Project administration, Methodology, Investigation, Conceptualization. **Eva Drevenhorn:** Writing – review & editing, Methodology, Investigation. **Helena Rosén:** Writing – review & editing, Writing – original draft, Project administration, Methodology, Investigation, Funding acquisition, Formal analysis, Conceptualization.

## Declaration of competing interest

No conflict of interest has been declared by any of the authors.

## Figures and Tables

**Table:** 

Date	Chosen flavour	Serving portions	Part of portion ingested in percentage	Other comments
				

## Appendix 1

### Diary for pilot study

Personal code number:

Register when the ice cream was eaten. The patient is offered 2 servings of 200 ml per day as a healthy food snack. Flavours to be chosen from (lemon, vanilla, caramel), the number of servings per day and the proportion of the servings that are eaten respectively drunk melted as a milkshake should be noted.

## Appendix 2

### Interview guide

Introductory question:

How did you perceive the protein-enriched ice cream?

Included questions:

What taste have you chosen?

How did you perceive the ice cream to be swallowing?

If you experience nausea, how is the ice cream to eat?

If you have burning in the mouth and throat, how is the ice cream to eat?

How do you perceive your satiety after eating a portion of ice cream?

Do you feel that you can improve your diet with the help of ice cream?

Do you wish to be able to choose any other flavour in addition to those available?

How do you perceive the ice cream when it is drunk as a cold “milkshake”? (the ice cream is thawed and served as a taste test in connection with the interview).
